# Supplementary material for: Development of a computer-aided design software for the quantitative evaluation of aesthetic damage
Source: PLoS One. 2019 Dec 18;14(12):e0226322. doi: 10.1371/journal.pone.0226322 (PMC6919621; doi:10.1371/journal.pone.0226322)
Supplement: S1 Code site — 1. (ZIP) [file pone.0226322.s001.zip › [EESC_jr][Projeto]Relatorio_07_21/reports/templates_zip/gentelella-master/vendors/Flot/examples/selection/index.html]

Flot Examples: Selection


## Selection

1000 kg. CO_2_ emissions per year per capita for various countries (source: Wikipedia).

Flot supports selections through the selection plugin. You can enable rectangular selection or one-dimensional selection if the user should only be able to select on one axis. Try left-click and drag on the plot above where selection on the x axis is enabled.

You selected:

The plot command returns a plot object you can use to control the selection. Click the buttons below.

Clear selection
Select year 1994

Selections are really useful for zooming. Just replot the chart with min and max values for the axes set to the values in the "plotselected" event triggered. Enable the checkbox below and select a region again.

Zoom to selection.

Copyright © 2007 - 2014 IOLA and Ole Laursen
